# Spirituality in Managing Perceived Stress and Promoting Self-Care: A Descriptive Study on Nursing Students in Spain

**DOI:** 10.1007/s10943-024-02232-z

**Published:** 2024-12-27

**Authors:** M. Dolores Fernández-Pascual, Abilio Reig-Ferrer, Ana M. Santos-Ruiz, Laura Martínez-Rodríguez

**Affiliations:** 1 Department of Health Psychology, Faculty of Health Sciences, University of Alicante, 03080 Alicante, Spain; 2 Institute for Health and Biomedical Research (ISABIAL), 03080 Alicante, Spain; 3 Fundamental and Clinical Nursing Department, Nursing Faculty, Universitat de Barcelona, Barcelona, Spain

**Keywords:** Nursing, Perceived stress, Self-care, Spirituality

## Abstract

The relationship between spirituality, perceived stress, and self-care was examined in a sample of 515 nursing students in Spain. Using the perceived stress scale (PSS), the professional self-care scale (PSCS), and the spirituality questionnaire (MiLS-sp/sf), the findings indicated that higher spirituality, particularly through inner peace and faith, was linked to reduced stress and enhanced self-care across physical, inner, and social dimensions. However, the inner self-care dimension was the least developed, suggesting that essential emotional, spiritual, and psychological needs were neglected. This result highlights the necessity for a comprehensive self-care model that empowers students to create personalised strategies to enhance their inner and spiritual self-care. It is essential that these findings give rise to practical applications in order to promote the well-being and professional effectiveness of nursing students.

## Introduction

### Self-Care in Nursing Practice

According to the main findings of the American Nurses Association Health Risk Appraisal study (ANA Health Risk Appraisal, 2017), nursing professionals with higher levels of personal well-being provide better patient care. Globally, several nursing codes of ethics include the requirement for self-care. These codes often incorporate the responsibility to protect and promote one's own health as part of the clearly defined obligation to provide quality patient care (Linton & Koonmen, 2020).

The International Council (ICN) Code of Ethics for Nurses (2012, 2021) identifies four main elements that provide a framework for ethical practice: nurses and patients or others requiring care or services; nurses and practice; nurses and the profession; and nurses and global health. Personal self-care is explicitly addressed in the case of the second component: nurses and practice. Specifically, it is recommended that nurses maintain a personal health level that does not compromise their ability to provide care.

### Self-Care Dimensions and Spirituality

Based the conceptual model proposed by Butler et al. (2019), self-care involves a conscious attentional process through intentional behaviours aimed at two general goals: a) protecting oneself and managing stress and other negative states and b) maintaining or improving personal well-being and overall functioning.

Six domains requiring the practice of self-care are defined: physical, professional, relational, emotional, psychological, and spiritual. Spiritual self-care, as conceptualised in this study, is derived from a broader understanding of spirituality such as a quest for meaning, purpose, and inner peace that may or may not be linked to religious beliefs (Puchalski et al., 2014). It encompasses practices that help individuals find meaning beyond themselves and foster a sense of connection to the larger world, whether through faith-based practices or secular experiences. This self-care dimension emphasises the cultivation of inner harmony and transcendence, which are essential to cope with stress and build resilience.

### Stress and Spiritual Well-Being in Nursing

Given the demands of their work, nursing professionals are particularly vulnerable to occupational stress, which significantly affects their personal well-being and the quality of the care they provide (Acea‐López et al., 2021; Torrente et al., 2021). In these high-intensity emotional situations which healthcare professionals frequently face, their ability to remain attentive, whole, and emotionally balanced is the greatest help they can offer. Indeed, this clarity, presence, and empathy allow them to provide constructive responses to the patients’ needs. And this self-awareness capacity not only allows for a higher level of self-care but also helps prevent emotional contagion while promoting resilience and professional satisfaction (Benito & Rivera, 2019).

A further analysis of the literature on the subject reveals that a relationship is emerging between spiritual well-being and stress (Nadifa et al., 2024; Yadav et al., 2017). Ample scientific evidence suggests that finding meaning in one’s life is positively and significantly associated with various measures of psychological well-being (e.g., happiness and life satisfaction), as well as with a substantial reduction in psychopathological risk (e.g., anxiety, depression, substance abuse, and suicide) (Debnam et al., 2018; Sakellari et al., 2018).

In addition, spiritual self-care has been identified as one of the resources that effectively helped healthcare staff manage the stress generated during the COVID-19 pandemic (Hawthorne & Barry, 2021).

### Focus on Nursing Students

Despite increasing recognition of the significance of spiritual well-being and self-care in managing stress within healthcare professions, comprehensive research on these relationships in nursing students remains limited. Most studies (Ausar et al., 2021) have focused on practicing healthcare professionals, leaving a research on how these dynamics affect students as they develop the skills and resilience required for their future roles.

Furthermore, the way self-care is conceptualised for nursing students varies considerably, contributing to inconsistencies in its integration into nursing education.

This variability complicates the ability of nursing educators to effectively support students in identifying their own self-care strategies and addressing systemic contributors to stress and burnout (Slemon et al., 2021).

The present study attempts to fill this gap by exploring how various self-care dimensions are related, including spiritual self-care and perceived stress in nursing students. By investigating this specific population, we aim to extend the literature on how spirituality and self-care practices may serve as essential resources for nursing students to manage their stress and well-being.

### Objectives

The evidence suggests the need to propose training initiatives to enhance self-awareness of care and the development of personal strategies, primarily regarding the spiritual dimension of care. Prior to designing the training action, we sought to address the following related issues:

- The determination of the nursing students’ levels of spiritual well-being, perceived personal self-care, and perceived stress.
- The relationship between spirituality, personal self-care dimensions, perceived stress, and relevant criterion variables.

## Methods

### Design

A cross-sectional study was conducted on a non-probabilistic convenience sample**.**

### Participants and Procedure

A convenience sampling method was used to include first-year nursing students from the University of Alicante, who participated during the second semester across three academic years (2021 to 2024). The inclusion criteria involved all 572 students enrolled in the first year of the programme and who were present during the data collection period. A total of 515 students attended on the data collection day and completed the study. Exclusion was based solely on attendance. Students who were absent in the practice session on the data collection day were excluded from the study. Table 1 displays the study participants' demographic information and two religiosity questions.

**Table 1:** Participants’ characteristics (N = 515)

Variable		
*Age*	Mean	SD
Years	20.43	5.83
*Gender*	Frequency	%
Female	405	78.6
Male	107	20.8
Non-binary	2	0.2
Prefer not to answer	1	0.4
*Degree of religious belief*		
Not religious	168	32.6
Slightly religious	227	44.1
Moderately religious	108	21.0
Very religious	12	2.3
*Belief in afterlife*		
No, nothing	105	20.4
There must be something	208	40.4
I think so	136	26.4
I'm sure of it	66	12.8

### Measures

#### Meaning in Life Scale (MiLS-sp/sf)

The Spanish adaptation (Reig-Ferrer et al., 2012, 2015) of the abbreviated version of the Meaning in Life Questionnaire (MiLS) evaluates a unique and coherent concept: spirituality as meaning in life.

A key consideration in selecting an appropriate measure of spirituality is the frequent conflation of spirituality and religiosity in existing scales. Many of these instruments do not effectively differentiate between the two constructs, potentially leading to biased interpretations of spirituality. In contrast, the meaning in life scale (MiLS) adheres to a widely accepted definition of spirituality, which emphasises an individual's quest to understand the meaning and purpose of their life. This perspective allows including religious beliefs and practices without positioning them as the central focus of the construct. By utilising the MiLS-sp/sf, our study aims to capture the essence of spirituality as a broader, more inclusive concept, facilitating a clearer understanding of its relationship with perceived stress and self-care among nursing students.

It explores four facets of meaning based on seven items described next. *Life Purpose* (items 1, 4) explores the degree of personal fulfilment, and life satisfaction currently felt by an individual based on their personal situation. It reflects engagement in activities, self-understanding, and optimism about the future. *Lack of Meaning* (items 2, 3) indicates a loss and decline in value of life and worth, as shown by a lack of motivation to do important things, a sense of confusion about oneself and life in general, and the belief that life is a negative experience. *Inner Peace* (items 5, 7) reveals the degree of internal peace and harmony, personal balance, inner experiences that make one feel good, and the presence of positive affectivity which provides tranquillity, serenity, and comfort. *Benefits of Spirituality* (item 6) assesses the degree of strength, fortitude, and comfort provided by religious faith or other spiritual beliefs independent of traditional religious systems. The instrument has demonstrated strong reliability, with an overall Cronbach's alpha of 0.86. Construct validity is supported by its correlations with subjective well-being measures documented in prior studies (Fernández-Pascual et al., 2020a, 2020b). The entire scale is presented in Table 4 (see Appendix).

#### Self-Care Scale

The professional self-care scale (PSCS; Galiana et al., 2015) is notably the only validated self-care scale available in Spanish specifically designed for healthcare professionals. Moreover, it is recognised as a valid and reliable tool for assessing self-care practices. This instrument evaluates self-care across three essential domains. The first is *physical self-care* (items 1, 2, and 5), which refers to implication in activities which help to maintain a healthy body. Factors such as getting enough sleep, eating properly, exercising frequently, and participating in healthcare and maintenance are some of the components that support good physical health.

The second is *inner self-care* (items 6, 7, and 8), which is related to mental well-being activities. Essential aspects of this care dimension include cultivating self-awareness and conscious reflection to achieve greater clarity and objectivity in personal experience, as well as engaging in recreational and enjoyable cognitive activities.

Third, *social self-care* (items 3, 4, and 9) encompasses social activities that contribute to maintaining social health. These relationships generally comprise a social network of people (close family and friends, coworkers, members of professional groups, or recreational activities) with whom we interact regularly, enriching our daily lives and whom we can turn to for emotional support in difficult situations.

The three dimensions constitute an overall self-care factor. The instrument is composed of nine items on a 5-point Likert scale (from 1, *“totally disagree”*, to 5, *“totally agree”*). The tool has been shown to be reliable, achieving an overall Cronbach's alpha of 0.80, with subscale values ranging from 0.78 to 0.81 across its three dimensions: physical, inner, and social self-care. The validity evidence aligns with previous research, confirming that it is suitable to apply to healthcare populations. The complete scale is presented in Table 5 (see Appendix).

#### Perceived Stress Scale (PSS)

The perceived stress scale, PSS (Cohen et al., 1983; Remor & Carrobles, 2001), is a 14-item self-report instrument with adequate psychometric properties that assesses the level of perceived stress over the past month and the extent to which individuals find their lives unpredictable, uncontrollable, or overloaded—key components repeatedly confirmed as central aspects of stress. The scale offers five response options: 0 = never; 1 = almost never; 2 = sometimes; 3 = often; and 4 = very often. The overall score is obtained by summing the values assigned to each item after converting the reverse-scored items (items 4, 5, 6, 7, 9, 10, 13). Higher total scores correspond to higher levels of perceived stress.

It was particularly relevant to select the PSS because of its strong psychometric properties and its validation across diverse populations, making it a favourite tool for perceived stress measurement. Notably, the PSS captures the subjective experience of stress, allowing for an in-depth understanding of how individuals interpret and react to stressors. This attribute is particularly helpful to examine the complex relationships between perceived stress, spirituality, and self-care practices, as it encompasses not only the frequency of stressors but also the personal meaning attributed to them. The Spanish version of the PSS presents even stronger applicability in this context, as it has shown robust psychometric properties, including an internal consistency of 0.81 and test–retest reliability of 0.73, as well as established concurrent validity and sensitivity (Remor, 2006).

Six additional variables were used as criteria to evaluate the student’s subjective well-being (health status, general quality of life, current quality of life, and personal happiness), as well as religious well-being (level of religiosity and belief in an afterlife). The question asked to evaluate the health status was: "In general, would you say your health is" (response options: Excellent; Very good; Good; Fair; Poor). To assess overall quality of life, the criterion question was: "Generally, would you say your quality of life is?" (response options: Very good; Good; Fair; Poor; Very poor). Item 9 from the COOP-WONCA questionnaire (Lizán-Tudela & Reig-Ferrer, 2002) was used to measure current quality of life: "How have things been going for you over the past 2 weeks?" (response options: Very well, could not be better; Pretty good; Sometimes good, sometimes bad. Good and bad equally; Pretty bad; Very bad, could not be worse). To evaluate personal happiness, the single-item question used was: "Generally speaking, would you say you are very happy, quite happy, not very happy, or not at all happy?".

All these well-being assessment variables have been proven to be valid and relevant in the specialised literature, and all these criterion-type questions had been analysed and tested by our group in previous research (Fernández-Pascual et al., 2017; Reig-Ferrer et al., 2012, 2015).

The scales used in this study were selected based on their validated psychometric properties and relevance to the constructs of spirituality, self-care, and perceived stress, and were reviewed to ensure they are not considered contaminated scales as defined by Koenig and Carey (2024).

### Data Collection

The self-assessment instrument was presented for self-completion in a single electronic document available on Moodle UA, the Moodle platform of the University of Alicante. The questionnaire was administered during a practical seminar at the beginning of the academic semesters over 3 consecutive years (2021–2022, 2022–2023, and 2023–2024). It was implemented well before the examination period, to ensure the latter would not potentially affect the students' psychological well-being and therefore the results.

### Ethical Considerations

Prior to completing the questionnaire, participants were informed about the study objectives and gave their informed consent regarding both their participation and the confidential handling of their data. They did not receive any financial or academic compensation for their involvement in the study, and no student refused to complete the questionnaire.

This study complied both with the ethical principles outlined in the Declaration of Helsinki (World Medical Association, 2024) and with national regulations, including the Spanish Organic Law 3/2018 on Personal Data Protection and Guarantee of Digital Rights (Ley Orgánica 3/2018, 2018). The study protocol was approved by the Ethics Committee of the University of Alicante (UA-2024-07-08_2).

### Data Analysis

The study data were analysed using the SPSS statistical software, version 28. Descriptive statistics were calculated to summarise the sociodemographic characteristics of the sample and the assessment questionnaire scores.

To improve its interpretability, the direct scores in the questionnaires were normalised on a scale from 0 to 10 using the Min–Max normalisation method (Han et al., 2012). This technique standardises the data on a scale from 0 to 10, making it possible perform direct comparisons across measures with different original ranges (e.g., 4-point, 5-point). The normalisation process involved subtracting the lowest possible value of the scale from each raw score, dividing by the range of the scale (highest value minus lowest value), and multiplying the result by ten. This approach ensures score interpretation consistency and facilitates comparisons between the study variables.

Additionally, correlational analyses using Pearson's correlation coefficient were conducted to explore the relationships between spirituality, perceived stress, and the self-care dimensions. Independent-sample t-tests were performed to assess potential gender differences in these variables.

## Results

**Fig. 1:**
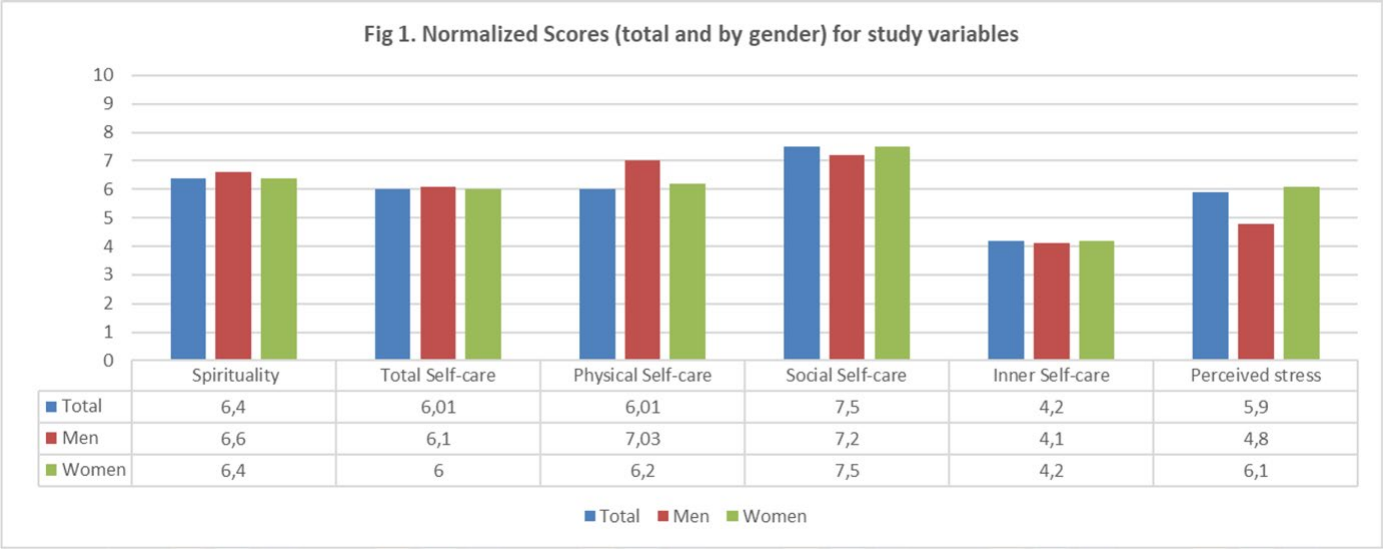
Normalised scores (total and by gender) for study variables

To address the first objective, the nursing students’ levels of spiritual well-being, perceived personal self-care, and perceived stress were analysed to establish a clear baseline for these variables. These findings are visually summarised in Fig. 1 through a bar chart, which presents the normalised scores, both total and by gender.

Accordingly, on a scale from 0 (indicating the lowest possible self-care score) to 10 (indicating the highest), the student sample demonstrated moderate-to-high averages (≥ 6) in two dimensions: Physical self-care (6.3) and social self-care (7.5), while inner self-care averages were notably lower (4.2). These results indicate that nursing students may be prioritising physical and social aspects of self-care, which play a crucial role in maintaining overall health and well-being. The significantly lower inner self-care average suggested a neglect of emotional and psychological needs compared to other self-care dimensions.

Gender differences were observed only in the Physical Self-care dimension (*t*(510) = 3.81, *p* = 0.001). Male students scored significantly higher than female students (Females: M = 10.41, DS = 2.53; Males: M = 11.44, SD = 2.28).

The normalised spirituality questionnaire average score was 6.4. The overall spirituality score did not present gender differences. However, upon closer inspection of individual items, significant differences were found in Item 3 (*t*(510) = 2.67, *p* = 0.001) and Item 6 (*t*(510) = 4.02, *p* = 0.001), where male students reported higher scores (i3: M = 5.19; SD = 0.97; i6: M = 4.36; SD = 1.12) compared to female students (i3: M = 4.89; SD = 1.25; i6: M = 3.85; SD = 1.20). The findings indicate that although men and women do not differ significantly in their overall spirituality levels, they may experience or express their spirituality in qualitatively different ways. Specifically, male students scored higher on items related to existential reflection and finding comfort in spiritual beliefs, suggesting that men may be focusing more on these spirituality aspects than women. These findings point to potential differences in how spirituality is experienced, even if global levels remained similar across genders.

The perceived stress scale (PSS) presented a mean score of 26.75, indicating high psychological stress levels among the study participants. Notably, 60% of the students scored above 24, i.e., a considerable share of the sample experienced substantial stress. A significant gender difference (*t*(510) = − 6.55, *p* = 0.001) was observed. Women reported a higher mean PSS score of 27.99 (SD = 8.02), which was statistically higher than men’s (22.28; SD = 8.05), thus highlighting gender disparity in perceived stress.

The relationships between spirituality, self-care dimensions, perceived stress, and criterion variables were then examined to address the second study objective.

Table 2 presents the relational behaviour of each personal self-care questionnaire (PSC) dimension with the items and total scores of the meaning in life scale (MiLS-sp) and the perceived stress scale (PSS).

**Table 2:** Relational analysis between the perceived stress scale, self-care dimensions, and items of the meaning in life scale

	PSS	PSC	ISC	SSC	TOTALPSCS
MiLS-sp/sf1	-.51**	.22**	.14**	.37**	.32**
MiLS-sp/sf2	.52**	-.20**	-.14**	-.37**	-.33**
MiLS-sp/sf3	.49**	-.23**	-.13**	-.33**	-.32**
MiLS-sp/sf4	-.18**	.23**	.19**	.17**	.29**
MiLS-sp/sf5	− .61**	.25**	.24**	.35**	.40**
MiLS-sp/sf6	-.69**	.27**	.27**	.36**	.42**
MiLS-sp/sf7	− .20**	.13**	.50**	.19**	.41**
MiLS-sp/sf Total	-.66**	.32**	.36**	.44**	.53**

MiLS-sp/sf: Meaning in Life Scale; PSS: Perceived Stress Scale; PSC: Physical self-care; ISC: Inner self-care; SSC: Social self-care; PSCS: Professional Self-Care Scale

^**^p < .01

The total spirituality score was positively and significantly associated with each self-care dimension. Spirituality appeared to play a particularly important role in the social self-care dimension.

A closer examination of key meaning in life scale items led to important insights. The total self-care score demonstrated robust correlations with specific dimensions of life’s meaning, particularly inner peace (items 5, 7) and benefits of spirituality (item 6). Specifically, the item reflecting a sense of balance within oneself (item 7) stood out as the facet of meaning that most strongly influenced the level of inner self-care in students. The latter suggests that those who feel more balanced are likely to engage in more effective self-care strategies.

The perceived stress scale (PSS) displayed a significant relationship with all meaning in life scale items (r = -0.66), the strongest correlations being observed in the case of faith in spiritual beliefs (r = − 0.69) and inner peace (r = − 0.61). These results underscore the importance of fostering a sense of inner peace and balance in students: indeed, not only are these factors linked to better self-care practices but they also play a crucial role in mitigating perceived stress.

The relational dynamics between the scales of subjective well-being and religious well-being variables are presented in Table 3.

**Table 3:** Bivariate analysis between the dimensions and total of self-care, the meaning in life scale, perceived stress scale, and the variables of subjective and religious well-being

	MiLS-sp/sf Total	PSS	PSC	ISC	SSC	TOTALPSCS
Belief in afterlife	.08	.11*	− .05	.23**	.03	.10*
Religious belief	.13**	.08	− .02	.31**	.06	.18**
General Quality of Life	.43**	− .51**	.25**	.15**	.38**	.36**
Current Quality of Life	.59**	− .66**	.20**	.12**	.35**	-.15**
Health Status	.40**	-.31**	.22**	.11*	.20**	.25**
Happiness	.50**	-.55**	.19**	.13**	.41**	.33**

MiLS-sp/sf: Meaning in Life Scale; PSS: Perceived Stress Scale; PSC: Physical self-care; ISC: Inner self-care; SSC: Social self-care; PSCS: Professional Self-Care Scale

^**^p < .01; *p < .05

The total spirituality score was positively and significantly associated with all self-reported subjective well-being variables: higher spirituality corresponded to better perceived health, higher self-reported quality of life, both overall and currently, and greater happiness as a person. This pattern was also observed in each of the three professional self-care scale dimensions and was inversely related to the total score on the perceived stress scale.

In relation to the religious well-being variables, a significant association was observed only with the Inner self-care scale of the professional self-care scale (PSCSS). Students who reported higher religiosity levels also demonstrated greater inner self-care.

## Discussion

A number of significant conclusions can be drawn from the findings as well as multiple discussion points, particularly regarding the interrelationships between the study variables.

First, the overall spirituality questionnaire scores suggest that the students’ meaning in life or spiritual well-being levels were satisfactory. These findings align with that of previous studies conducted by our research group (Fernández-Pascual et al., 2018, 2020a, 2020b; Reig-Ferrer et al., 2019). This nursing student group’s level of perceived and manifested spiritual well-being was significantly higher than that of other Spanish patient groups, such as those with cancer, renal diseases (Reig-Ferrer et al., 2012), and those in palliative care (Reig-Ferrer et al., 2015).

Regarding the self-care scale results, the student sample scored highest in the social dimension of care, followed closely by the physical dimension. However, the inner dimension of care showed significantly lower scores.

This finding may reflect a lack of training or education on the importance of inner self-care practices. Such a gap could hinder the development of effective coping strategies in stressful environments, emphasising the need for a more comprehensive understanding of self-care that incorporates emotional and psychological dimensions.

Our findings are consistent with the literature. In the review conducted by Younas (2017), nursing students were found to be aware of the importance of maintaining a balanced diet, sleep hygiene, and a level of physical activity to improve their physical health. However, they tended to neglect self-care practices that could enhance their emotional, psychological, and spiritual health due to academic stress, workload, and inadequate knowledge about self-care improvement strategies in these domains. Such barriers have also been documented in other recent studies (Ausar et al., 2021; Brouwer et al., 2021).

The fact that healthcare professionals, including nursing students, are particularly vulnerable to stress is well-established. This vulnerability often emerges well before entering professional practice, specifically during their academic training. Prior research has extensively documented this phenomenon in first-year nursing students (Jones & Johnston, 1997, 1999), identifying them as a higher risk group regarding stress than students in other fields. Previous reviews (Arian et al., 2023; Labrague et al., 2017; McCarthy et al., 2018) have concluded that nursing students generally experience moderate-to-high levels of negative stress due to factors such as patient care responsibilities, academic assignments, workload, negative interactions with staff, perceived clinical incompetence, and examinations. In this study, 76.5% of the students experienced acute negative stress. This finding is in line with similar international studies which have also reported high levels of stress in university students (Al-Zayyat & Al-Gamal, 2014; Blomberg et al., 2014; Hamaideh et al., 2017; Toqan et al., 2023; Zhao et al., 2015).

Consistent with previous studies indicating that women tend to report higher perceived stress levels than men (Graves et al., 2021), our findings also demonstrated significant gender differences in perceived stress.

Concerning the connection between the scales and criterion variables, our study was compatible with other findings in the literature (Arora & Mandal, 2023; Borges et al., 2021; Chiang et al., 2021; De Diego-Cordero et al., 2022; Mahamid & Bdier, 2021). The relational analysis confirmed evidence of convergent validity: Spirituality and self-care were positively and significantly associated with perceived health, personal happiness, and quality of life, while being negatively associated with perceived stress.

These findings present significant implications for nursing education and the enhancement of student well-being.

Notable is the fact that integrating self-care practices—particularly spiritual well-being practices—into the nursing curriculum could potentially foster resilience and improve the students’ capacity to manage both academic and clinical stressors. The existing literature has demonstrated that spirituality and self-care practices are instrumental in enhancing emotional resilience and promoting a positive outlook on life (McGuinness & Nordstokke, 2023; Schwalm et al., 2022; Yun et al., 2019).

Furthermore, the negative correlation observed between these practices and perceived stress underscores their role as protective factors, aiding individuals in coping with academic and personal pressures (Ma, 2023). Consequently, educators should prioritise a more flexible and individualised approach, encouraging students to engage with self-care practices that resonate with their personal values and unique needs. A recent mapping review of nurses' self-care strategies highlights the diverse ways in which self-care can be implemented, reinforcing the necessity of tailored approaches to personal variations in these practices (Gantt & Haberstroh, 2023).

### Limitations

The study limitations give rise to future lines of research. First, the use of a non-probabilistic convenience sampling method restricted the generalisability of the findings to the wider population of nursing students. This approach may introduce selection bias due to participant availability, as well as institutional bias, given that the sample was drawn from a single university.

Second, the cross-sectional and correlational nature of the research design did not allow for causal inferences. Future studies incorporating a longitudinal design could offer more robust insights into the temporal dynamics and causal relationships between the variables examined.

## Conclusions

Based on the above findings, it is crucial to integrate self-care, particularly spiritual well-being, into nursing education curricula. Moving forward, educational institutions should consider adopting tailored interventions which help students to identify and cultivate their own self-care practices, rather than relying on a one-size-fits-all approach. Specifically, spiritual well-being, which has been shown to correlate with reduced stress levels and improved overall well-being, should be incorporated as an essential component of nursing training.

Concrete implementation steps could include the embedding of reflective practices, mindfulness exercises, and opportunities for students to explore and discuss the role of spirituality in both personal and professional contexts.

In addition, educators can play a pivotal role in supporting students' mental health by leading workshops and activities that encourage the development of individualised self-care strategies. Collaborating with students to co-create these strategies could foster a more inclusive and empowering learning environment, where students are encouraged to take ownership of their mental and emotional health.

Future research should also focus on evaluating the long-term impact of these curricular interventions on students' professional well-being and stress management, ensuring that the benefits extend beyond their academic careers and into their professional lives.

## Appendix

See Tables 4, 5.

**Table 4:** MiLS-sp/sf questionnaire items

MiLS-sp/sf1	I feel more fulfilled and satisfied with life
MiLS-sp/sf2	My life has less meaning
MiLS-sp/sf3	I do not value my life as much as before
MiLS-sp/sf4	I have found new goals that are more worthwhile
MiLS-sp/sf5	I feel at peace with myself
MiLS-sp/sf6	I find strength and comfort in my faith or spiritual beliefs
MiLS-sp/sf7	I have a sense of balance within myself

**Table 5:** Professional self-care scale items

1P	I do exercise on a regular basis
2P	I usually follow a balanced diet
3S	I believe that my relations outside work are satisfactory
4S	I believe that my family relations are satisfactory
5P	I practice activities that help me to relax
6I	My self-care includes getting actively involved in spiritual practice, meditation, oration…
7I	I am constant. I have continuity with my spiritual practice
8I	When I feel emotionally overloaded I try to find time for my own care
9S	When I feel overwhelmed by a clinical situation I feel that I can support on my team in order to elaborate this experience
